# Case Report: Fatal prosthetic valve endocarditis due to *Staphylococcus lugdunensis*—a wolf in coagulase-negative clothing

**DOI:** 10.3389/fmed.2026.1704784

**Published:** 2026-02-03

**Authors:** Raj Patel, Ekta Patel, Riddhi Patel

**Affiliations:** 1 Lake Erie College of Osteopathic Medicine, Erie, PA, United States; 2 Mt. Sinai Morningside Hospital, New York, NY, United States

**Keywords:** antimicrobial therapy, case report, coagulase-negative *Staphylococcus*, prosthetic valve endocarditis, *Staphylococcus lugdunensis*

## Abstract

*Staphylococcus lugdunensis* is a rare but highly virulent coagulase-negative *Staphylococcus* (CoNS) capable of causing aggressive native and prosthetic valve endocarditis. Unlike other CoNS, it resembles *S. aureus* in its ability to rapidly destroy cardiac structures and cause systemic complications. We present a woman in her 70s with a prosthetic mitral valve who initially presented with altered mental status and was diagnosed with *S. lugdunensis* prosthetic valve endocarditis. Despite targeted intravenous antibiotics, her condition deteriorated rapidly, and she unfortunately died within 5 days of admission. This case highlights the need to distinguish *S. lugdunensis* from other CoNS and to recognize its aggressive course and limited response to antibiotics alone.

## Introduction

Prosthetic valve endocarditis (PVE) remains a serious and often fatal complication of valve replacement surgery; these cases are associated with a high risk of structural valve destruction, systemic embolization, and mortality (1). CoNS are frequently isolated pathogens in late-onset PVE, generally regarded as low-virulence skin commensals and contaminants in blood cultures. However, *S. lugdunensis* represents a clinically significant exception.

Though classified as a CoNS, *S. lugdunensis* exhibits virulence characteristics more similar to *S. aureus*, including rapid endothelial invasion, cytotoxin production, and the ability to form destructive vegetation (2). Recent data demonstrate that *S. lugdunensis* bacteremia carries a higher risk of infective endocarditis (IE) compared to other CoNS, with an aggressive course and clinically significant mortality (3). IE caused by *S. lugdunensis* accounts for 1% of all IE cases, but these cases can have up to 40% mortality (4). Timely recognition is crucial, as suboptimal antibiotic therapy is associated with poor outcomes, and surgical intervention is often required due to rapid valvular damage and hemodynamic compromise (5).

We report a fatal case of *S. lugdunensis* PVE in a woman with a remote history of prosthetic mitral valve replacement. She presented with an atypical presentation of subacute altered mental status and was diagnosed with PVE based on the modified Duke major and minor clinical criteria. Despite early initiation of targeted antibiotic therapy, she rapidly deteriorated and was deemed a poor surgical candidate due to prohibitive perioperative risk. She ultimately died from multiorgan failure and septic shock within 5 days of admission. This case highlights the diagnostic challenge of a rare but aggressive pathogen, which should not be dismissed as a contaminant, and the therapeutic challenge of managing this pathogen in patients who are not surgical candidates.

## Case description

The patient was a female in her 70s with a history of dementia, infective endocarditis s/p mitral valve replacement with Edwards Magna tri-leaflet bovine pericardium bioprosthetic valve 4 years prior to the current visit, hypertension, hyperlipidemia, and iron deficiency anemia, who presented to the emergency department for a 1-week history of altered mental status. Due to dementia, the patient was unable to provide a reliable history, but collateral information obtained revealed that the patient had been feeling unwell for the past week. She had a non-productive cough, decreased oral intake, shortness of breath, chest pain, palpitations, fever, chills, night sweats, headache, dizziness, imbalance, diplopia, tinnitus, and urinary burning/urgency/frequency. According to the family, she was getting more confused as the week went on, but it was initially attributed to her dementia. However, on the day of the ED visit, she was found in bed acutely altered with slurred speech, an episode of incontinence, right arm numbness and tingling, and generalized weakness. History also revealed that she had a fall a few days prior, secondary to the loss of balance, and she hit her head. She does not have a personal history of stroke or a family history of neurological disorders. On a physical exam, the patient did not have a fever, a new heart murmur, signs of heart failure, or any of the classic peripheral signs of infective endocarditis, such as Janeway lesions, Osler nodes, or splinter hemorrhages.

Given the chief complaint of altered mental status with her history of dementia, an appropriate neurological workup was conducted, which revealed numerous hypodense areas concerning stroke on CT head and scattered acute/subacute infarcts involving bilateral cerebral and cerebellar hemispheres on brain MRI (Supplementary Figure 1). Subsequent MR angiography did not reveal any significant stenosis.

The patient met clinical criteria for SIRS on admission, presenting with tachycardia, tachypnea, and significant leukocytosis (14.28), with a pro-calcitonin level of 2.74 supporting suspicions. SIRS protocol was initiated, and subsequent workup failed to reveal any acute processes or significant findings on imaging, suggesting an infectious etiology. Blood cultures drawn showed grown Gram-positive cocci, later determined to be *S. lugdunensis*, and the patient was empirically started on IV vancomycin 2 g once, piperacillin-tazobactam 4.5 g once, and ceftriaxone 2 g daily.

The patient quickly became hemodynamically unstable and declined into septic shock. She developed multi-organ dysfunction, including acute kidney injury (creatinine of 1.72 from a baseline of 0.80), cardiac demand ischemia (troponins consistently around 100), pulmonary edema, and thrombocytopenia. Her mental status was fluctuating; she was able to answer some questions and had improved neurologically since admission, but she still had periods of altered mental status. Due to her clinical status, she was ultimately transferred to the ICU.

In the ICU, the patient required multiple vasopressors to maintain adequate tissue perfusion. Repeated blood cultures since admission showed persistent *S. lugdunensis* bacteremia, and she was switched to IV cefazolin 2 g q12h. The addition of gentamicin was considered but was not given due to her worsening renal function. On ICU night 1, she started to have multiple episodes of cardiac arrhythmias, and laboratory values continued to deteriorate. A transthoracic echocardiogram showed elevated mitral valve peak gradient and velocity with concerns for infective endocarditis (Supplementary Figure 2), and a subsequent transesophageal echocardiogram revealed an obstructive, highly echogenic, and mobile mass attached to the medial mitral leaflet with extension into the periprosthetic area and the inter-valvular fibrosa, confirming the suspicion of prosthetic valve endocarditis and explaining her new arrhythmogenic status (Supplementary Figure 3).

The cardiothoracic surgery team was consulted for surgical evaluation, but the patient was deemed not a surgical candidate due to prohibitive perioperative risk. At this point, the options were either to continue medical management or to transition to palliative care. Her laboratory values continued to deteriorate; her WBC count was up to 24.41, creatinine up to 4.32, AST up to 3,623, and lactic acid at 4.0. Palliative care followed up with the patient, and she decided not to forego resuscitation or intubation if required. She was transitioned to comfort care, and all life-sustaining medications were discontinued. Unfortunately, the patient’s condition deteriorated too rapidly, and she passed away 5 days after admission.

## Discussion

*S. lugdunensis* is a CoNS that is increasingly recognized as a highly virulent pathogen capable of causing fulminant IE, including PVE. CoNS are often dismissed as blood culture contaminants, but unlike typical CoNS such as *S. epidermidis*, *S. lugdunensis* possesses aggressive virulence properties such as biofilm formation, cytolysins, metalloproteases, hemolysins, adhesins, and heme-uptake iron sequestration proteins (4). Due to these properties, *S. lugdunensis* is associated with aggressive clinical presentations, including large vegetations, valve perforation, rapid hemodynamic decline, and abscess formation, leading to higher mortality rates compared to IE from other CoNS (6).

Our patient’s clinical course (altered mental status, constitutional symptoms, and rapid progression to multiorgan failure) mirrors the abrupt and destructive nature of *S. lugdunensis* PVE. While *S. lugdunensis* typically remains susceptible to beta-lactams such as oxacillin and cefazolin, empiric therapy often includes vancomycin until definitive identification and susceptibility profiles are available (2). Our patient was empirically started on piperacillin-tazobactam, ceftriaxone, and vancomycin and later switched to cefazolin after susceptibility results were obtained, consistent with the recommended antibiotics for coverage of *S. lugdunensis.* However, despite receiving early antibiotic therapy, her condition deteriorated rapidly, highlighting the inadequacy of antibiotic therapy alone in cases of severe valvular destruction.

Current guidelines from the American Heart Association and European Society of Cardiology recommend early surgical intervention in IE complicated by heart failure, persistent bacteremia, embolic risk reduction, or PVE, many of which our patient had (1). For *S. lugdunensis* IE, surgery is often essential rather than optional due to the organism’s destructive potential. The landmark study by Anguera (5), though older, demonstrated better outcomes among *S. lugdunensis* IE patients who underwent valve replacement versus those treated with medical therapy alone.

This case contributes to the growing body of evidence highlighting the virulence of *S. lugdunensis* in the setting of prosthetic valve infections, which should be treated as a true pathogen and differentiated from other CoNS when isolated from blood cultures. Its marked virulence underscores the importance of early microbiological identification, prompt initiation of targeted antimicrobial therapy, and aggressive infection management. However, despite optimal antibiotic treatment, the prognosis remains poor in cases of severe valvular involvement without surgical intervention. These situations highlight the need for early goals-of-care discussion, including palliative care involvement, given the high likelihood of rapid clinical deterioration.

## Data Availability

The original contributions presented in the study are included in the article/Supplementary material, further inquiries can be directed to the corresponding author.
