# Supplementary material for: Case Report: Fatal prosthetic valve endocarditis due to Staphylococcus lugdunensis—a wolf in coagulase-negative clothing
Source: Front Med (Lausanne). 2026 Feb 3;13:1704784. doi: 10.3389/fmed.2026.1704784 (PMC12910833; doi:10.3389/fmed.2026.1704784)
Supplement: Supplementary file 1 [file Data_Sheet_1.pdf]

Supplemental Material

The supplemental material for this case report includes (1) multiple axial views of the patient's MRI brain to illustrate the diffuse bilateral infarcts suspicious of a septic etiology, (2) a transthoracic echocardiogram illustrating the large vegetation on the medial mitral leaflet, and (3) transesophageal echocardiogram illustrating the large vegetation on the mitral valve and the spontaneous echo contrast in the left atrium from the pooling of blood.

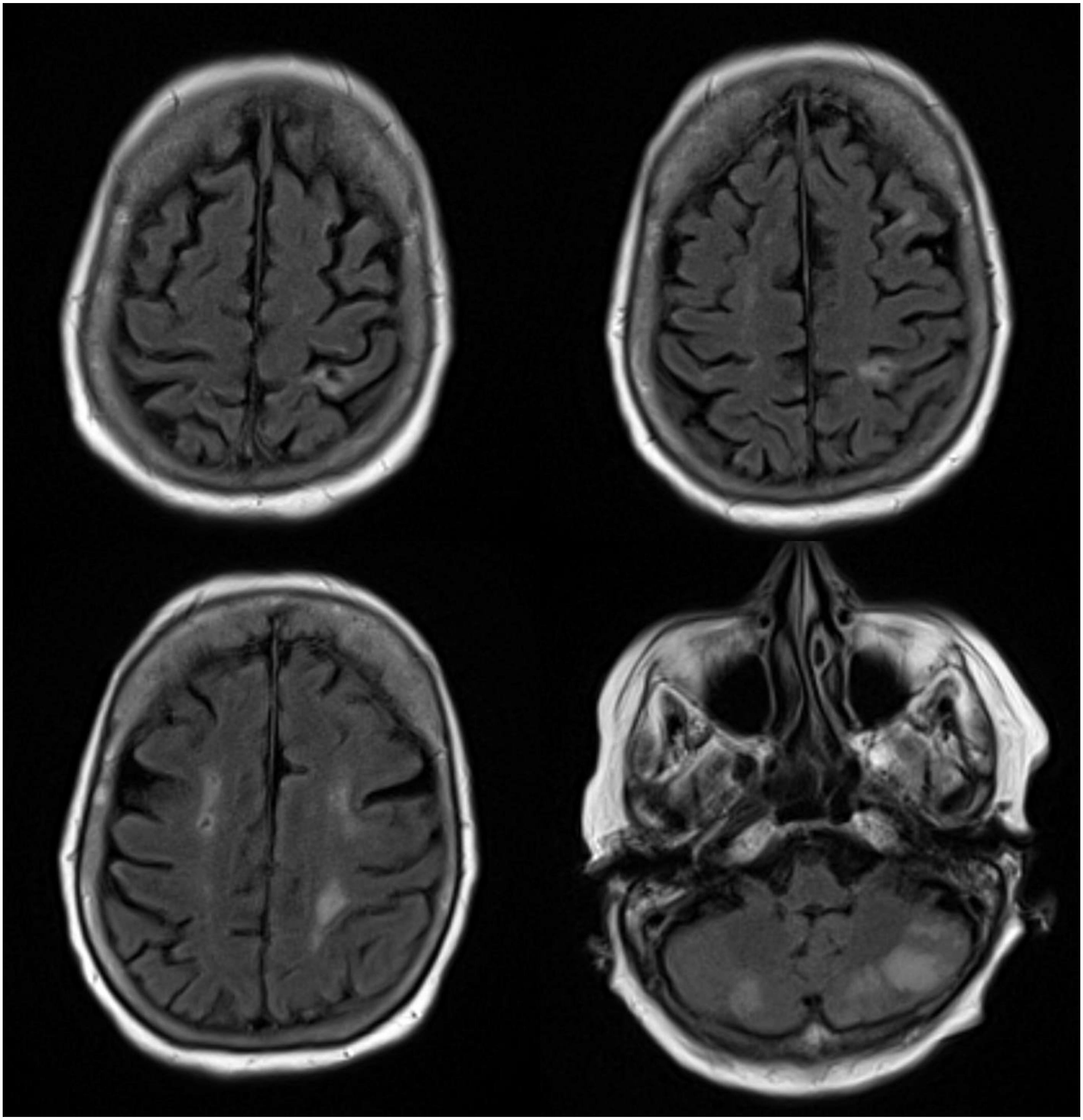

Supplemental Figure 1: Various transverse views of brain MRI showing diffuse bilateral cerebral and cerebellar infarcts presumed to be from septic emboli

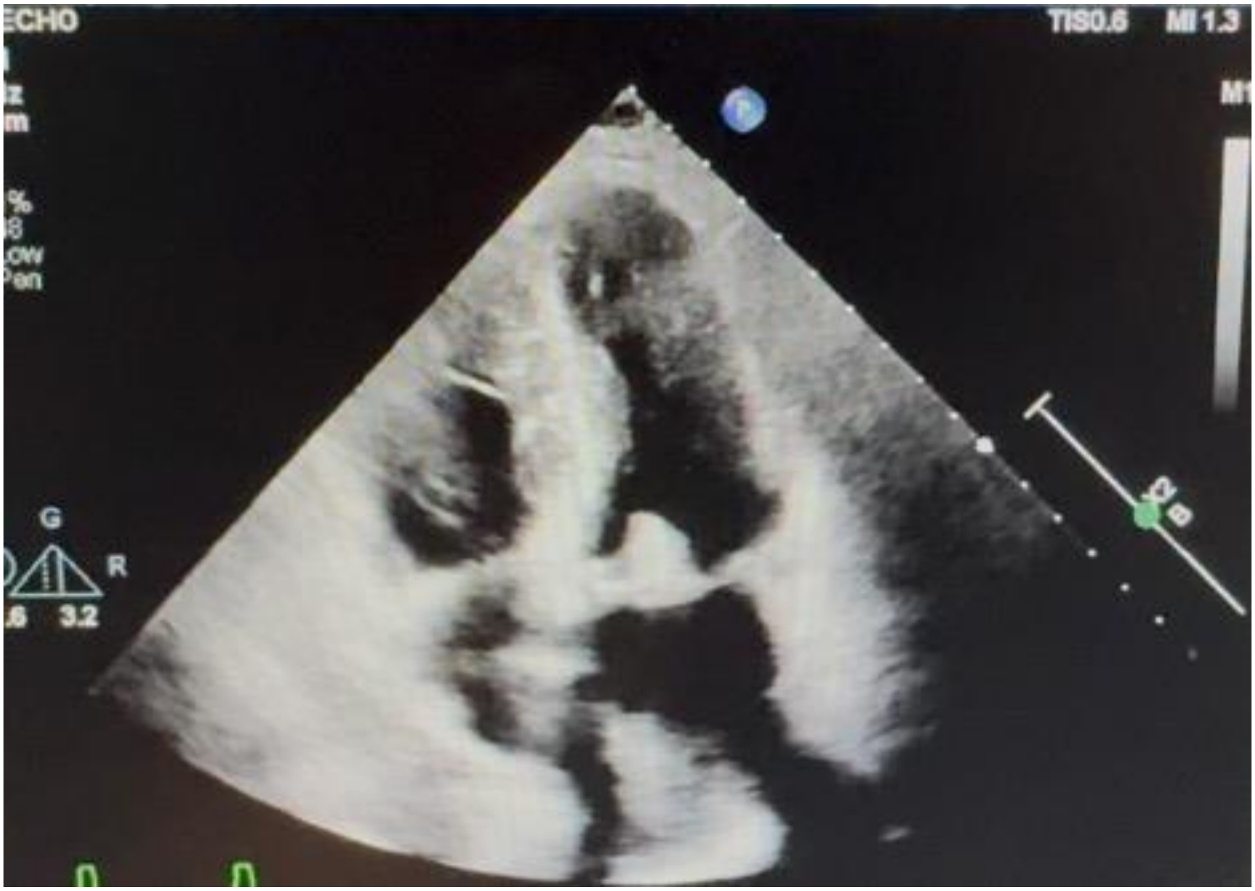

Supplemental Figure 2: Transthoracic echocardiogram showing large vegetation on medial mitral leaflet

A)

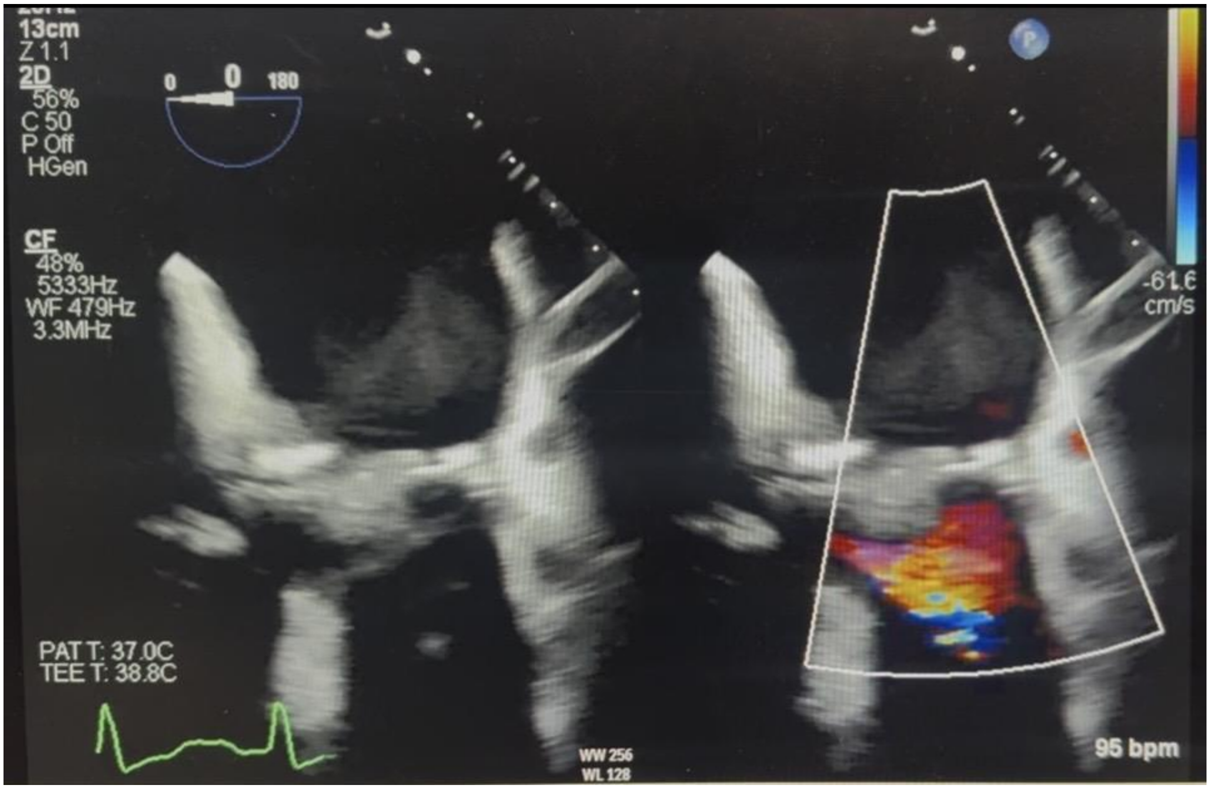

B)

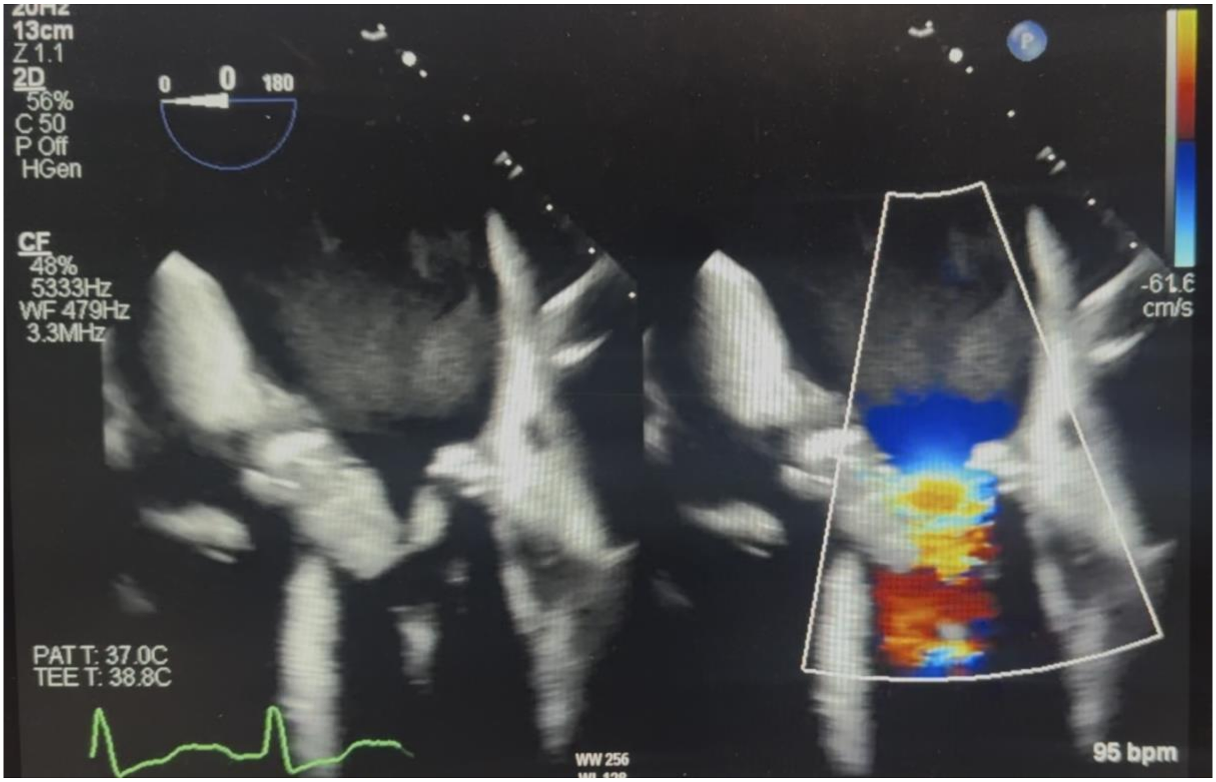

Supplemental Figure 3: Transesophageal echocardiogram with Doppler in systole (A) and diastole (B) showing large medial mitral valve vegetation with extension into the periprosthetic area and intervalvular fibrosa and spontaneous echo contrast in left atrium
